# Spontaneous cholecystocutaneous fistula as a rare presentation of advanced gallbladder adenocarcinoma: a case report and review of the literature

**DOI:** 10.1097/RC9.0000000000001104

**Published:** 2026-10-15

**Authors:** Ahmed vadel Jidou, Oussama Douhi, Manal Derkaoui, Rachid Jabi, Amal Bennani, Mohammed Bouziane

**Affiliations:** a Department of General Surgery, Mohamed VI University Hospital, Oujda, Morocco; b Faculty of Medicine and Pharmacy, Laboratory of Anatomy, Microsurgery and Surgery Experimental and Medical Simulation (LAMCESM), Mohammed First University, Oujda, Morocco; c Department of Pathology, Mohamed VI University Hospital, Faculty of Medicine and Pharmacy of Oujda, Oujda, Morocco

**Keywords:** case report, cholecystocutaneous fistula, chronic cholecystitis, gallbladder adenocarcinoma, spontaneous biliary fistula

## Abstract

**Introduction and importance:**

Spontaneous cholecystocutaneous fistula (SCCF) is an exceptionally rare complication of gallbladder disease in the modern era and is only rarely associated with gallbladder carcinoma. Its atypical clinical presentation may delay diagnosis and treatment.

**Case presentation:**

A 79-year-old woman from a rural area presented with a fistulized abdominal wall abscess evolving for 3 months. Her medical history included type two diabetes mellitus, left lower-limb amputation due to diabetic foot disease, and thrombectomy for right popliteal artery occlusion. Physical examination revealed an indurated cutaneous fistula with inflammatory changes over the right flank. Laboratory investigations showed mild anemia and elevated inflammatory markers, while liver function tests were within normal limits. Contrast-enhanced thoraco-abdomino-pelvic computed tomography demonstrated irregular thickening of the gallbladder infiltrating hepatic segment V with omental extension, associated with an air-fluid collection that communicated with the abdominal wall and peritoneal cavity, consistent with a SCCF. Histopathological examination confirmed advanced gallbladder adenocarcinoma.

**Discussion:**

SCCF has become extremely uncommon owing to advances in imaging, antibiotic therapy, and early surgical management of biliary disease. While most reported cases are secondary to neglected cholelithiasis, fistulization caused by gallbladder carcinoma remains exceptional. Chronic inflammation, tumor necrosis, and direct local invasion are considered the principal mechanisms involved. This unusual presentation may mimic a chronic abdominal wall abscess, potentially delaying recognition of the underlying malignancy.

**Conclusion:**

SCCF may represent a rare presenting manifestation of advanced gallbladder adenocarcinoma. Underlying malignancy should be considered in elderly patients presenting with atypical abdominal wall abscesses or biliary-cutaneous fistulas. Cross-sectional imaging and histopathological examination are essential for establishing the diagnosis and guiding management.

## Introduction

Spontaneous cholecystocutaneous fistula (SCCF) is an exceptionally rare complication of gallbladder disease, defined as an abnormal tract between the gallbladder and the skin. Once more frequently encountered in the pre-antibiotic era, SCCF is now extremely uncommon in contemporary surgical practice due to advances in early diagnosis and timely management of biliary disease, including the widespread use of ultrasonography and laparoscopic cholecystectomy^[^1,2^]^.

HIGHLIGHTS

- Spontaneous cholecystocutaneous fistula is an exceptionally rare complication of gallbladder disease in the modern era.
- Gallbladder adenocarcinoma may rarely present as a cutaneous fistula, delaying diagnosis and treatment.
- Cross-sectional imaging plays a crucial role in identifying the fistulous tract and the underlying malignancy.
- Histopathological examination is essential for establishing the diagnosis of gallbladder adenocarcinoma.
- Clinicians should consider occult gallbladder cancer in patients presenting with spontaneous biliocutaneous fistulas.

The majority of biliary cutaneous fistulas arise as a late complication of chronic cholelithiasis and recurrent cholecystitis. Persistent cystic duct obstruction may lead to gallbladder distension, ischemia, and eventual perforation, resulting in localized abscess formation that can spontaneously drain through the abdominal wall^[^3^]^. In most cases, these fistulas develop gradually and may present as chronic draining sinuses, often misdiagnosed as simple soft tissue infections or abscesses, particularly in elderly or immunocompromised patients^[^4^]^.

Although benign gallstone disease remains the most common etiology, gallbladder carcinoma is a rare but important underlying cause of SCCF. Gallbladder cancer is a highly aggressive malignancy, frequently diagnosed at an advanced stage due to its nonspecific clinical presentation and anatomical location^[^5^]^. Local invasion into adjacent hepatic parenchyma, the peritoneum, and abdominal wall structures may lead to fistula formation, particularly when tumor necrosis and secondary infection are present^[^6^]^. Such presentations are exceptionally uncommon and often indicate advanced, unresectable disease.

Several authors have highlighted that malignant biliary cutaneous fistulas are frequently preceded by a prolonged inflammatory process, during which clinical and radiological findings may mimic benign gallbladder pathology^[^7^]^. This overlap contributes to delayed diagnosis and poor outcomes. Cross-sectional imaging, particularly contrast-enhanced computed tomography (CT) and magnetic resonance imaging (MRI), plays a central role in identifying gallbladder wall irregularity, soft tissue infiltration, and fistulous tracts^[^8^]^. However, definitive diagnosis relies on histopathological confirmation, which remains essential for distinguishing between benign inflammatory disease and malignancy^[^9^]^.

From a pathophysiological standpoint, SCCF formation in gallbladder carcinoma is thought to result from a combination of tumor necrosis, chronic infection, and progressive erosion through adjacent tissues, eventually reaching the skin surface^[^10^]^. Because of its rarity, there are no standardized management guidelines, and treatment strategies depend primarily on the underlying cause and stage of the disease. While benign cases are managed surgically with cholecystectomy and fistula excision, malignant cases often carry a poor prognosis and require individualized oncological management^[^11^]^.

The patient underwent a biopsy of the lesion. Histopathological examination demonstrated an infiltrating moderately differentiated adenocarcinoma.

This work has been reported following the SCARE Guidelines 2025^[^12^]^.

No AI tools were used in the research and development of the manuscript.

## Case presentation

The patient was a 79-year-old woman from a rural area who was admitted with a fistulized parietal abdominal wall abscess that had evolved over 3 months. Her medical history included type two diabetes mellitus diagnosed 5 years earlier and treated with oral antidiabetic agents. She had previously undergone a left lower-limb amputation in 2021 for diabetic foot disease, although medical records were not available. She also had a history of a right popliteal artery occlusion treated surgically by thrombectomy, followed by antiplatelet therapy with clopidogrel; documentation was unavailable.

On clinical examination, the patient was conscious and hemodynamically stable, with a World Health Organization performance status of 3–4. She was anicteric. Abdominal examination revealed midline laparotomy scars, both supra- and infra-umbilical. There was an indurated, inflamed cutaneous fistula located over the right flank (Fig. 1).

**Figure 1.**
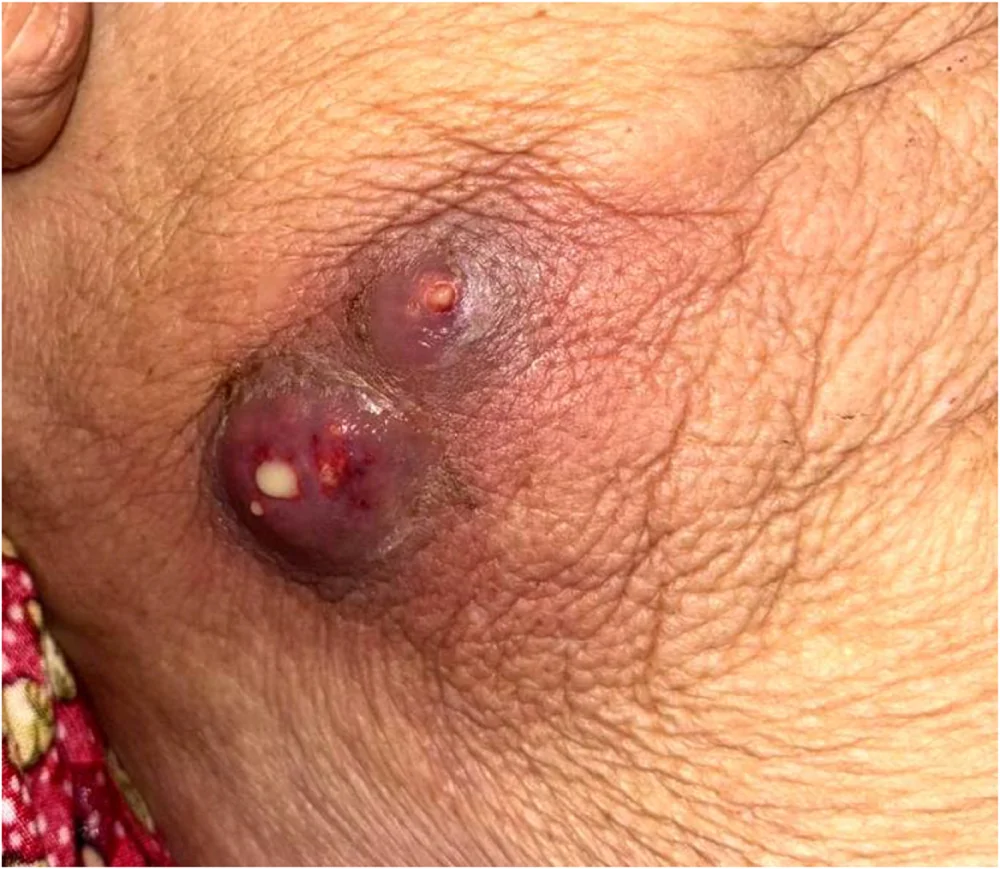
Clinical image showing a cholecystocutaneous fistula.

Biological investigations showed mild anemia, with a hemoglobin level of 10.4 g/dL, and elevated inflammatory markers, with a C-reactive protein level of 58 mg/L. Liver function tests were within normal limits.

Contrast-enhanced thoraco-abdomino-pelvic CT scan demonstrated irregular parietal thickening of the gallbladder body infiltrating hepatic segment V, associated with omental involvement. An air-fluid collection was identified within the right abdominal wall musculature, communicating with both the intraperitoneal cavity and the subcapsular hepatic region, consistent with a fistulizing process. A small amount of ascites was also noted (Fig. 2).

**Figure 2.**
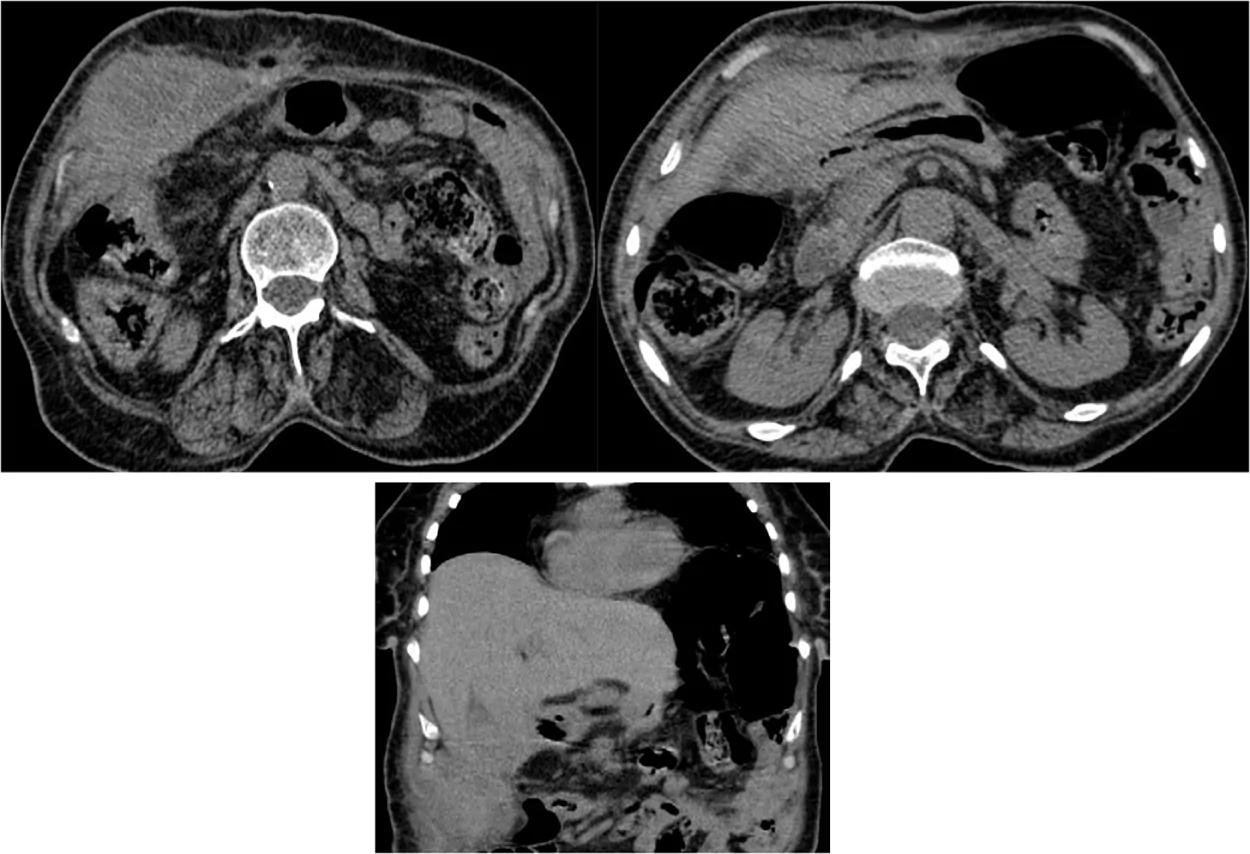
Computed tomography images showing irregular thickening of the gallbladder wall involving the body of the gallbladder, with extension into hepatic segment V and associated omental infiltration. A hydroaeric collection within the right abdominal wall musculature communicates with the peritoneal cavity and the subcapsular hepatic space, suggesting a fistulized process secondary to locally advanced disease.

The patient subsequently underwent a biopsy of the lesion. Histopathological examination revealed a moderately differentiated adenocarcinoma. Immunohistochemical analysis demonstrated tumor cell positivity for CK7 and CK19, and negative staining for CK20, supporting a pancreatobiliary origin and consistent with primary gallbladder adenocarcinoma (Fig. 3).

**Figure 3.**
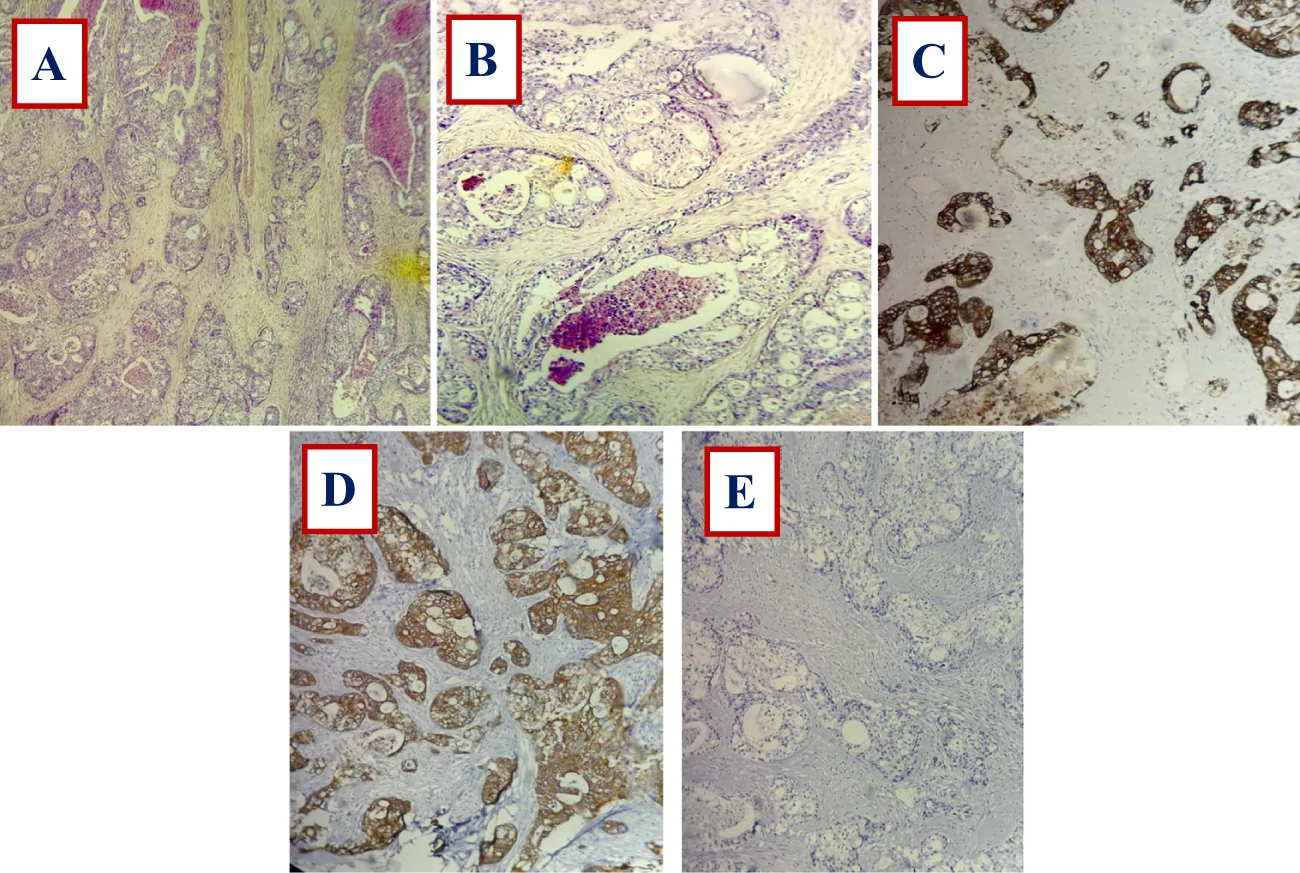
Histopathological images. (A) Infiltrating malignant glandular proliferation composed of irregular glands lined by atypical cells with vacuolated cytoplasm, dissecting an abundant desmoplastic stroma, consistent with a moderately differentiated gallbladder adenocarcinoma (H&E × 10). (B) H&E × 20. (C) Positive immunostaining for CK7 in tumor cells. (D) Positive immunostaining for CK19 in tumor cells. (E) Negative immunostaining for CK20 in tumor cells.

The patient was referred to the medical department for further evaluation and possible medical treatment.

## Discussion

SCCF is an extremely uncommon complication of gallbladder disease in the current era, largely due to advances in early imaging, broad-spectrum antibiotics, and timely cholecystectomy. Historically, external biliary fistulas were described more frequently in the pre-antibiotic era, but today they are considered rare clinical entities, most often associated with neglected gallstone disease. In contrast, fistulization secondary to gallbladder carcinoma is exceptional and only sporadically reported in the literature^[^13,14^]^.

Pathophysiology and mechanisms of fistula formation:

The formation of a SSCF is generally the result of chronic inflammation leading to gallbladder wall necrosis, perforation, and adherence to adjacent structures. In benign disease, prolonged cholelithiasis with recurrent episodes of cholecystitis results in progressive erosion of the gallbladder wall. The inflamed gallbladder may become adherent to the parietal peritoneum and anterior abdominal wall, eventually forming a tract that drains externally^[^15^]^.

In malignant disease, the mechanism differs slightly. Gallbladder adenocarcinoma can induce fistulization through a combination of tumor necrosis, rapid local invasion, and ischemic degeneration of the gallbladder wall. The tumor may directly invade adjacent hepatic parenchyma, omentum, and abdominal wall structures, creating a path of least resistance for drainage. This mechanism is associated with advanced-stage disease and reflects a locally aggressive biological behavior^[^16^]^.

Epidemiology and clinical presentation:

Gallbladder carcinoma is the most common biliary tract malignancy but remains relatively rare globally. Its incidence shows marked geographic variation, being higher in certain regions such as South America and South Asia. The disease predominantly affects elderly patients, shows a female predominance, and is frequently diagnosed at an advanced stage due to nonspecific symptoms or incidental discovery after cholecystectomy^[^9^]^.

SCCF typically presents as a chronic, non-healing abdominal wall abscess or a discharging sinus. This atypical presentation can lead to misdiagnosis as a simple soft tissue infection, especially in patients with diabetes mellitus or immunosuppression. In the present case, the lesion mimicked a chronic, infected abdominal wall abscess, delaying suspicion of an underlying hepatobiliary malignancy.

Imaging findings and diagnostic approach:

Cross-sectional imaging plays a central role in the evaluation of suspected SCCF. Contrast-enhanced CT is particularly useful in identifying gallbladder wall thickening, local invasion, hepatic infiltration, and fistulous tracts. In malignant cases, CT may reveal irregular gallbladder wall thickening, loss of fat planes between the gallbladder and liver, and associated soft tissue collections extending toward the abdominal wall^[^17^]^.

MRI and MR cholangiopancreatography can provide additional detail regarding biliary anatomy and fistulous communication, although CT remains the most widely used initial modality because of its availability and speed.

In the present case, CT imaging demonstrated irregular gallbladder wall thickening with invasion of hepatic segment V and extension into the abdominal wall musculature, along with a hydro-aeric collection communicating with the peritoneal cavity and subcapsular hepatic region. These findings were highly suggestive of a fistulized malignant process.

Histopathology and immunohistochemistry:

Histopathological examination remains the gold standard for definitive diagnosis. Gallbladder carcinoma is most commonly an adenocarcinoma, often moderately to poorly differentiated at diagnosis. The tumor in this case was a moderately differentiated adenocarcinoma, consistent with typical histological patterns described in the literature.

Immunohistochemistry is essential for confirming the pancreatobiliary origin of the tumor and excluding metastatic disease. An immunoprofile of CK7-positive and CK19-positive, with CK20 negativity, is characteristic of primary biliary tract adenocarcinomas, including gallbladder carcinoma. CK7 and CK19 are commonly expressed in epithelial cells of the biliary tree, whereas CK20 is more frequently associated with colorectal and other lower gastrointestinal malignancies. This pattern strongly supports a primary gallbladder origin in the present case^[^18,19^]^.

### Differential diagnosis

The differential diagnosis of a chronic draining abdominal wall lesion is broad and includes infected epidermoid cysts, chronic osteomyelitis with sinus formation, actinomycosis, complicated diverticular disease, Crohn’s disease with fistulization, and post-surgical fistulas. In elderly patients with risk factors such as diabetes mellitus and vascular disease, infectious etiologies are often initially favored. However, persistent or atypical lesions should prompt evaluation for underlying malignancy, particularly when imaging reveals deep tissue involvement or hepatobiliary abnormalities.

### Management considerations

The management of SCCF depends primarily on the underlying etiology and the stage of disease. In benign cases related to gallstones, treatment typically involves control of infection followed by elective cholecystectomy and excision of the fistulous tract. However, in malignant cases, management is more complex and often limited by advanced disease at presentation.

Surgical resection, including extended cholecystectomy with partial hepatectomy, may be considered in selected patients with localized disease. Unfortunately, most cases of gallbladder carcinoma that present with fistulization are diagnosed at an advanced stage, making curative resection unlikely. Palliative management, including drainage of infected collections and systemic therapy, is often the mainstay of treatment^[^20^]^.

### Prognosis

The prognosis of gallbladder carcinoma remains poor, with overall 5-year survival rates below 10% in most series. Prognosis is strongly dependent on stage at diagnosis, with significantly better outcomes in early-stage disease. The presence of fistulization indicates locally advanced disease with tissue invasion and is generally associated with an unfavorable prognosis^[^9^]^.

### Clinical implications

This case underscores the importance of considering hepatobiliary malignancy in the differential diagnosis of chronic or atypical abdominal wall infections. Elderly patients presenting with persistent fistulizing lesions should undergo prompt imaging evaluation. Early recognition of this rare presentation may allow earlier diagnosis and more appropriate therapeutic decision-making.

## Conclusion

SCCF is an exceptionally rare clinical presentation in the modern era and may occasionally reveal an underlying malignancy. In this case, it represented the initial manifestation of advanced gallbladder adenocarcinoma with local invasion. Because clinical presentation can mimic a chronic abdominal wall abscess, diagnosis may be delayed, particularly in elderly patients with multiple comorbidities. This case highlights the importance of maintaining a high index of suspicion for biliary tract malignancy in patients presenting with atypical abdominal wall fistulas or SCCF. Cross-sectional imaging plays a crucial role in identifying the underlying hepatobiliary origin and assessing local extension, but definitive diagnosis relies on histopathological and immunohistochemical confirmation. Early recognition of this rare entity is essential to guide appropriate management and improve diagnostic accuracy in such complex presentations.

## Data Availability

Not applicable.

## References

[1] LeBlanc KA Barr LH Rush BM. Spontaneous biliary enteric fistulas. South Med J 1983;76:1249–52. 10.1097/00007611-198310000-00013 6623136

[2] Roslyn J Busuttil RW. Perforation of the gallbladder: a frequently mismanaged condition. Am J Surg 1979;137:307–12. 10.1016/0002-9610(79)90056-4 434321

[3] Niemeier OW. Acute free perforation of the gall-bladder. Ann Surg Juin 1934;99:922–4. 10.1097/00000658-193499060-00005 PMC1390061 17867204

[4] Giles AE Godzisz S Nenshi R. Diagnosis and management of acute cholecystitis: a single-centre audit of guideline adherence and patient outcomes. Canadian J Surg Juin 2020;63:E241–9. 10.1503/cjs.002719 PMC7828997 32386475

[5] Randi G Franceschi S La Vecchia C. Gallbladder cancer worldwide: geographical distribution and risk factors. Int J Cancer 2006;118:1591–602. 10.1002/ijc.21683 16397865

[6] Halaseh SA Halaseh S Shakman R. A review of the etiology and epidemiology of gallbladder cancer: what you need to know. Cureus 2022;14:e28260. 10.7759/cureus.28260 PMC9491243 36158346

[7] Deng X Yuan YC Tian W. Gallbladder cancer masquerading as xanthogranulomatous cholecystitis: a case report and literature review. Front Oncol 2024;14. doi: 10.3389/fonc.2024.1409347 PMC11288967 39087023

[8] MD UT and KR. Globalradcme. Gallbladder carcinoma. Disponible sur. 2021. Accessed 20 June 2026. https://www.globalradiologycme.com/single-post/gallbladder-carcinoma

[9] Hundal R Shaffer EA. Gallbladder cancer: epidemiology and outcome. Clin Epidemiol 2014;6:99–109. 10.2147/CLEP.S37357 PMC3952897 24634588

[10] Thomas MB. Biological characteristics of cancers in the gallbladder and biliary tract and targeted therapy. Crit Rev Oncol Hematol 2007;61:44–51. 10.1016/j.critrevonc.2006.07.006 17164111

[11] Goetze TO. Gallbladder carcinoma: prognostic factors and therapeutic options. World J Gastroenterol 2015;21:12211–7. 10.3748/wjg.v21.i43.12211 PMC4649107 26604631

[12] Revised Surgical CAse REport (SCARE) guideline: an update for the age of Artificial Intelligence - premier science. Disponible sur. 2025. Accessed 16 September 2025. https://premierscience.com/pjs-25-932/

[13] Flora HS Bhattacharya S. Spontaneous cholecystocutaneous fistula. HPB (Oxford) 2001;3:279–80. 10.1080/136518201753335584 PMC2020630 18333032

[14] Glenn F Reed C Wr G. Biliary enteric fistula. Surgery, gynecology & obstetrics. 1 December 1957. Accessed 20 June 2026. Disponible sur. https://www.semanticscholar.org/paper/Biliary-enteric-fistula.-Glenn-Reed/0f6ad361fa2728cbb0a3b5e49179a3099de3b414

[15] Quiroga-Garza A Alvarez-Villalobos NA Angeles-Mar HJ. Localized gallbladder perforation: a systematic review of treatment and prognosis. HPB 2021;23:1639–46. 10.1016/j.hpb.2021.06.003 34246546

[16] Ha GW Lee MR Kim JH. Cholecystocolic fistula caused by gallbladder carcinoma: preoperatively misdiagnosed as hepatic colon carcinoma. World J Gastroenterol 2015;21:4765–9. 10.3748/wjg.v21.i15.4765 PMC4402327 25914489

[17] Oikarinen H Päivänsalo M Lähde S. Radiological findings in cases of gallbladder carcinoma. Eur J Radiol 1993;17:179–83. 10.1016/0720-048x(93)90100-2 8293745

[18] Offerhaus G. Tumors of the gallbladder, extrahepatic bile ducts and ampulla of vater. atlas of tumor pathology. J Clin Pathol 2001;54:816.

[19] Chu P Wu E Weiss LM. Cytokeratin 7 and cytokeratin 20 expression in epithelial neoplasms: a survey of 435 cases. Mod Pathol 2000;13:962–72. 10.1038/modpathol.3880175 11007036

[20] Hueman MT Vollmer CM Pawlik TM. Evolving treatment strategies for gallbladder cancer. Ann Surg Oncol Août 2009;16:2101–15. 10.1245/s10434-009-0538-x 19495882

